# Sleep problems in preschool children at the child development center with different developmental status: A questionnaire survey

**DOI:** 10.3389/fped.2022.949161

**Published:** 2022-09-14

**Authors:** Chi-Man Kuok, Jia-Rou Liu, Jao-Shwann Liang, Shao-Han Chang, Ming-Tao Yang

**Affiliations:** ^1^Department of Pediatrics, Far Eastern Memorial Hospital, New Taipei, Taiwan; ^2^Child Development Center, Far Eastern Memorial Hospital, New Taipei, Taiwan; ^3^Department of Nursing, Asia Eastern University of Science and Technology, New Taipei, Taiwan; ^4^Taiwan International Graduate Program in Interdisciplinary Neuroscience, National Cheng Kung University and Academia Sinica, Taipei, Taiwan; ^5^Institute of Biomedical Sciences, Academia Sinica, Taipei, Taiwan; ^6^Department of Chemical Engineering and Materials Science, Yuan Ze University, Taoyuan, Taiwan

**Keywords:** developmental disabilities, sleep wake disorders, preschool child, premature birth, gestational age

## Abstract

**Objective:**

To investigate the relationship between sleep problems and development in preschool children with suspected developmental delay.

**Methods:**

A total of 192 preschool children (mean age 4 years; 131 males, 61 females) were recruited from the Child Development Clinic, including 98 preterm children and 94 age- and sex-matched full-term children. All participants underwent evaluation of gross motor, fine motor and speech performance. All parents of all participants completed the Children's Sleep Habits Questionnaire (CSHQ). Some of the participants also underwent psychological evaluation. Correlation analysis and community network analysis were used to investigate the interactions.

**Results:**

The developmental status was: 75.5% developmental delay, 19.3% borderline development, and 5.2% normal development. Eighty-nine percent of the subjects had abnormal CSHQ scores. Age, gestational age, speech development, cognitive development, and socio-emotional development were significantly correlated with the CSHQ. Significant interactions between sleep problems and development were noted mostly in the preterm group.

**Conclusion:**

High prevalence of sleep disturbances in children at the Child Development Center was noted and associated with multiple factors. Therefore, during the multidisciplinary evaluation of children with possible developmental delay, inquiring about their sleep quality and habits is strongly recommended. Mitigating sleep problems enhances the efficacy of early intervention programs.

## Introduction

Sleep problems are common in children, especially in those with developmental disabilities and those born prematurely (1, 2). Sleep problems are also attributable to developmental problems in children (3), and the risk of preterm birth in mothers (4). Neurological disability, cognitive impairment, and sleep problems may affect children's school performance.

Sleep problems are prevalent, long-lasting, and complex in children with developmental delay (2). Sleep problems in specific neurodevelopmental disorders have been previously reviewed (5). Common sleep problems in children with attention deficit/hyperactivity disorder (ADHD) include short sleep duration, delayed sleep onset, and bedtime resistance. Sleep anxiety, delayed sleep onset, bedtime resistance, increased night waking, and shorter sleep duration are common in children with autism spectrum disorder (ASD). In children with cerebral palsy, airway obstruction with hypertonia leads to sleep-disordered breathing (SDB), and increased night waking. Excessive daytime sleepiness, increased night waking, and SDB are common in children with Down syndrome.

Poor sleep also affects children's performance and health (3). Interestingly, poor sleep worsens attention deficit in children with ADHD, but increases ADHD-trait behaviors in children with typical development (6). In addition, a short nighttime sleep duration was associated with lower cognitive and language scores at 2 years of age, and persistent SDB was associated with lower language scores (3). Sleep-dependent learning in early childhood has been proposed for the influence of sleep on neurodevelopment (7).

Preterm children are at an increased risk of neurological disabilities and cognitive impairment (8). They also experience more sleep problems than the full-term children, as indicated by parent-report sleep problems and objective equipment including polysomnography, electroencephalogram, and actigraphy (1). Common sleep problems in preterm children include longer sleep durations, earlier bedtimes and wake times, difficulties initiating or maintaining sleep, night waking, and SDB (9, 10). These sleep problems correlated with attention problems and negative emotionality in preterm children (10). However, few studies have focused on sleep problems of preschool/younger children born prematurely. In addition, it has not been explored well-whether developmental delay aggravates sleep problems in preterm preschool children.

This study thus aimed to investigate the relationship between sleep problems and development in preschool children with suspected developmental delay, and to determine whether gestational age affects these interactions.

## Materials and methods

### Participants

Preschool children were recruited from the Child Development Clinic at the Child Development Center in Far Eastern Memorial Hospital, a medical center in North Taiwan, between January 1 and December 31, 2021. All children suspected of having developmental delay were referred to the Child Development Clinic for formal evaluation. Children born prior to 37 weeks of gestation were included in the preterm group. Based on gestational age, preterm birth was sub-categorized as extremely preterm (<28 weeks), very preterm (28–32 weeks), moderate preterm (32–34 weeks), and late preterm (34–37 weeks) (11). Age- and sex-matched full-term children were denoted as the full-term group.

The exclusion criteria were major congenital anomalies and parents who were unwilling to participate in this study.

### Developmental evaluation

All evaluations are overseen by a developmental pediatrician, child psychiatrist, and pediatric physiatrist during the multidisciplinary team assessment. All participants (*n* = 192) underwent developmental evaluation of their gross motor, fine motor and speech performance. Some of the participants also received psychological evaluation depending on the child psychiatrist's impression; only those children (*n* = 127, 66%) suspected to have psychiatric problem were referred for psychological evaluation. Motor development was assessed using the Peabody Development Motor Scales second edition for children younger than 6, and the Movement Assessment Battery of Children second edition for children aged 6. Speech and language development were assessed using the Communication and Language Screening Test for 0–3-year-old children and the Preschool Language Scale for 3–6-year-old children. Cognitive development was assessed using the Bayley Scales of Infant Development third edition for 0–3-year-old children, and the Wechsler Preschool and Primary Scale of Intelligence for 4–7-year-old children. ADHD was diagnosed through a diagnostic interview by child psychiatrists and psychologists, the Kindergarten Children's Activity Rating Scale for children aged >3 years, the Attention-Deficit/Hyperactivity Disorder Test for children aged >4 years, and Conners' Kiddie Continuous Performance Test for 4–7-year-old children. ASD was diagnosed through a diagnostic interview by child psychiatrists and psychologists, the Clancy Behavior Scale for 2–5-year-old children, and the Childhood Asperger's Syndrome Test for children aged ≥4. Referring to age-matched norms, each development domain was diagnosed as normal, borderline, or delayed.

### Children's sleep habits questionnaire

The parents also completed the Children's Sleep Habits Questionnaire (CSHQ), a traditional Chinese version, during the participants' developmental evaluation. The CSHQ is the most commonly used screening tool to identify sleep problems in children (12). It contains 35 parent-report questions grouped into eight subscales: (1) bedtime resistance, (2) sleep onset delay, (3) sleep duration, (4) sleep anxiety, (5) night wakings, (6) parasomnias, (7) sleep-disordered breathing, (8) daytime sleepiness. Each item is rated on a 3-point Likert-type scale that describes the frequency of the behaviors. After eliminating 2 duplicate items, 33 items yield a total sleep disturbance score. A cut-off total sleep disturbance score of 41 yields a sensitivity of 0.80 and specificity of 0.72 for sleep problem (12).

### Community network analysis

In community networks analysis, we analyzed the strength of interactions between the sleep problems and developmental status in the full-term and preterm groups. The coherence coefficient (r) between two *X*_*a*_and *X*_*b*_ indexes were calculated by the Spearman correlation coefficient method, presented by edges, and labeled with the values of correlation coefficients and significant *p*-values. The community network was calculated and illustrated by the open resource JASP (available at: https://jasp-stats.org/).

### Statistical analysis

A paired *t*-test was used to compare age and CSHQ scores between the full-term and preterm groups. Pearson's chi-squared test was used to compare sex and developmental status between these two groups. Spearman's rank correlation test was used to measure the strength of the association between developmental status and CSHQ scores. All data were analyzed using IBM^®^ SPSS^®^ Statistics software version 19.0 (IBM Inc., Somers, NY, USA). All tests were two-tailed and a *p-*value <0.05 was considered statistically significant.

## Results

### Participants' epidemiological and developmental status

**Figure 1:**
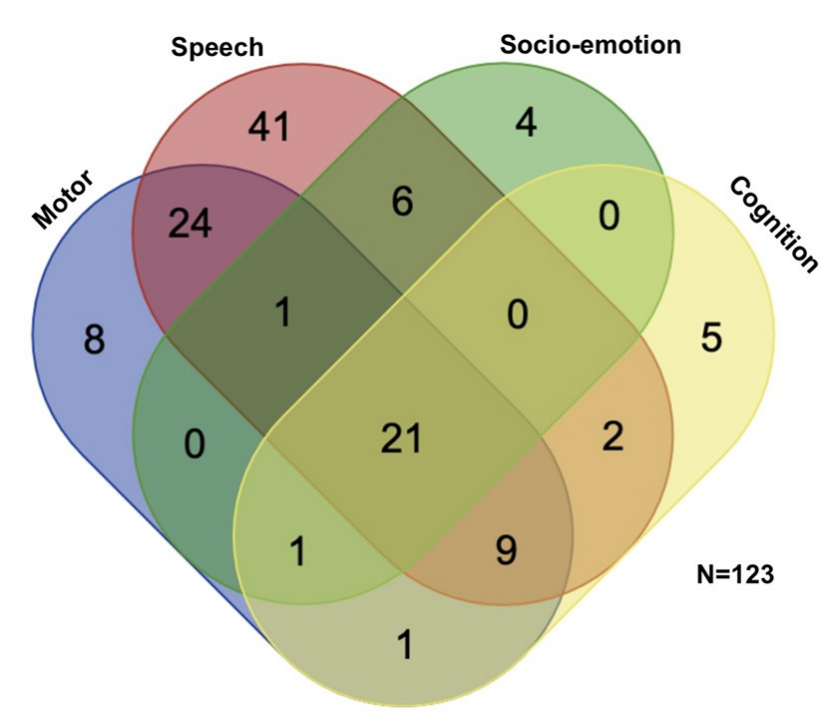
Participants' distribution of different domains of neurodevelopmental delay. Selecting the most common four domains of developmental delay, more than half (*n* = 65, 53%) of the children with developmental delay had mixed developmental delay. Speech developmental delay was the most prevalent (*n* = 104, 85%), followed by motor developmental delay (*n* = 65, 53%), cognitive developmental delay (*n* = 39, 32%), and socio-emotional delay (*n* = 33, 27%).

A total of 192 children (mean age 4 years; 131 males, 61 females) suspected to have developmental delay were enrolled in this study (Table 1). Ninety-eight children were enrolled in the preterm group, 70.4% of whom were late preterm. The control group comprised 94 age- and sex-matched full-term children. The ratio of boys to girls was 2 to 1. All participants underwent evaluation of gross motor, fine motor and speech development; Only some of the children (*n* = 127, 66%), suspected to have psychiatric problem, underwent psychological evaluation. In total, 145 (75.5%) children had developmental delay, 37 (19.3%) had borderline development, and only 10 (5.2%) had normal development. Among developmental delay, speech developmental delay was the most common. We selected the common developmental delay (motor, speech, cognition, and socio-emotional; *n* = 123) for presenting isolated and mixed developmental delay (Figure 1). More than a half (53%) of our participants had mixed developmental delay. There were no significant differences in age, sex, or developmental status between the full-term and preterm groups. There were no significant differences in age, sex, or gestational age between children with developmental delay and those with normal/borderline development.

**Table 1:** Epidemiological and neurodevelopmental status of the subjects.

	**Total**	**Full-term**	**Preterm**	***P*-value**	**NDBD**	**DD**	***P*-value**
*N*	192	94	98		47	145	
Male/Female	131/61	67/27	64/34	0.374	31/16	100/45	0.700
Age (year)	4.00 ± 1.53	4.03 ± 1.51	3.96 ± 1.56	0.748	4.26 ± 1.55	3.91 ± 1.52	0.167
Full-term							0.464
Term		43 (45.7%)			13	30	
Early		51 (54.3%)			12	39	
Preterm							0.521
Late			69 (70.4%)		17	52	
Moderate			14 (14.3%)		1	13	
Very			8 (8.2%)		2	6	
Extremely			7 (7.1%)		2	5	
Neurodevelopment (N/B/D)	10/37/145	5/20/69	5/17/76	0.780	10/37/0	0/0/145	**<0.001**
Gross motor	69/85/38	31/46/17	38/39/21	0.444	27/24/0	42/61/38	**<0.001**
Fine motor	104/35/53	53/17/24	51/18/29	0.796	45/6/0	59/29/53	**<0.001**
Speech	76/11/105	36/6/52	40/5/53	0.892	46/5/0	30/6/105	**<0.001**
Comprehension	89/11/92	42/7/45	47/4/47	0.589	50/1/0	39/10/92	**<0.001**
Expression	84/13/95	42/6/46	42/7/49	0.957	47/4/0	37/9/95	**<0.001**
Cognition	69/17/39	36/11/16	33/6/23	0.241	31/2/0	38/15/39	**<0.001**
Socio-emotion	64/26/34	32/13/18	32/13/16	0.958	19/10/0	45/16/34	**0.001**
ADHD	65/31/31	39/14/12	26/17/19	0.111	16/15/0	49/16/31	**<0.001**
Autism	95/7/23	45/4/15	50/3/8	0.291	29/1/0	66/6/23	**0.007**

Age is presented as mean ± standard deviation. All other data are presented as the number of participants or number (%). ADHD, attention deficit-hyperactivity disorder; N/B/D, normal/borderline/delay; DD, developmental delay; NDBD, normal development/borderline development. Bold font indicates statistical significance.

### Sleep problem of the participants

Using a cut-off total sleep disturbance score of 41 (12), a total of 172 (89.6%) participants in this study were categorized as abnormal, indicating a high risk for sleep problems (Table 2). Full-term children and children with developmental delay had higher rates of sleep problems than preterm children and children with normal or borderline development, although the differences were not significant. Only the sleep onset delay subscale showed significant differences, and bedtime resistance showed marginal differences between the full-term and preterm groups (Table 2). Parents reported that full-term children experienced more sleep onset delay and bedtime resistance problems than preterm children. The other CSHQ subscales showed no differences between these two groups. All CSHQ scores showed no differences between children with developmental delay and those with normal/borderline development, even in the preterm group.

**Table 2:** CSHQ scores of the subjects.

								**Preterm**		
	**Total**	**Full-term**	**Preterm**	***P*-value**	**NDBD**	**DD**	***P*-value**	**NDBD**	**DD**	***P*-value**
*N*	192	94	98		47	145		22	76	
Sleep problem (%)	172 (89.6)	85 (90.4)	87 (88.8)	0.708	40 (85.1)	132 (91.0)	0.248	18 (81.8)	69 (90.8)	0.260
Total sleep disturbance score	49.17 ± 7.66	50.07 ± 7.27	48.31 ± 7.95	0.110	50.15 ± 8.21	48.86 ± 7.48	0.315	48.36 ± 8.27	48.29 ± 7.91	0.970
Bedtime resistance	10.94 ± 1.90	11.20 ± 1.86	10.69 ± 1.92	0.064	11.15 ± 2.01	10.88 ± 1.87	0.394	10.91 ± 2.09	10.63 ± 1.88	0.553
Sleep onset delay	1.76 ± 0.76	1.88 ± 0.80	1.63 ± 0.69	0.022^*^	1.74 ± 0.77	1.76 ± 0.76	0.913	1.50 ± 0.51	1.67 ± 0.74	0.221
Sleep duration	4.10 ± 1.26	4.18 ± 1.20	4.02 ± 1.32	0.379	4.02 ± 1.15	4.12 ± 1.30	0.628	3.73 ± 0.88	4.11 ± 1.41	0.133
Sleep anxiety	7.43 ± 2.17	7.66 ± 1.98	7.21 ± 2.32	0.155	7.64 ± 2.33	7.37 ± 2.11	0.454	7.64 ± 2.77	7.09 ± 2.17	0.403
Night wakings	4.19 ± 1.43	4.26 ± 1.52	4.13 ± 1.34	0.553	4.15 ± 1.23	4.21 ± 1.49	0.810	4.14 ± 1.28	4.13 ± 1.36	0.988
Parasomnia	9.30 ± 2.23	9.40 ± 2.08	9.20 ± 2.37	0.535	9.36 ± 2.39	9.28 ± 2.18	0.833	9.09 ± 2.81	9.24 ± 2.24	0.801
Sleep disordered breathing	3.57 ± 0.85	3.57 ± 0.84	3.56 ± 0.86	0.914	3.60 ± 0.77	3.56 ± 0.87	0.795	3.68 ± 0.84	3.53 ± 0.87	0.459
Daytime sleepiness	12.40 ± 3.23	12.66 ± 3.00	12.15 ± 3.44	0.279	13.02 ± 3.39	12.20 ± 3.17	0.130	12.00 ± 3.16	12.20 ± 3.53	0.814

Sleep problem is presented as number (%). All other data are presented as mean ± standard deviation. CSHQ, the Children's Sleep Habits Questionnaire; DD, developmental delay; NDBD, normal development/borderline development.

* *p* < 0.05. Bold font indicates statistical significance.

### Correlation between epidemiological and developmental status and the CSHQ

**Figure 2:**
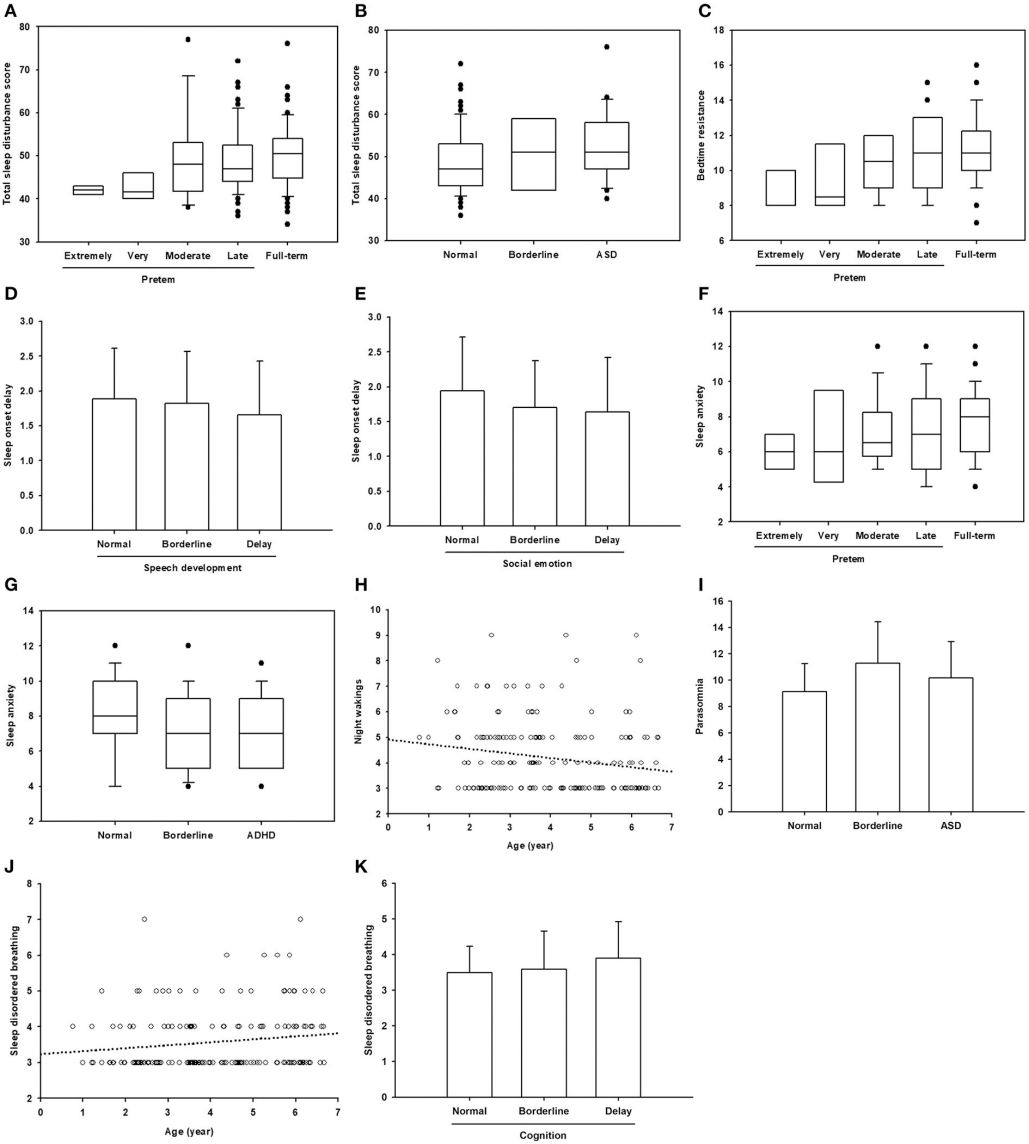
Significant correlations between age, gestational age, and neurodevelopmental status with sleep problems. **(A–C,F,G)** Data are presented in box plot. Within each box, a horizontal line denotes median value. Boxes extend from the 25th to the 75th percentile of values. Dots denote outliers. **(D,E,I,K)** Data are presented as mean ± standard deviation. **(H,J)** The circles represent the plotted values while the line represents the best fit for the correlation between the two variables.

An analysis of the correlation between epidemiological and developmental status and the CSHQ (Table 3; Figure 2) found that age, gestational age, speech development, cognitive development, socio-emotional development, ADHD, and ASD were significantly correlated with the CSHQ. The total sleep disturbance score was positively correlated with gestational age and ASD (Figures 2A,B). Similarly, bedtime resistance was positively correlated with gestational age (Figure 2C). Sleep onset delay was negatively correlated with speech developmental and socio-emotional delay (Figures 2D,E). Sleep anxiety was positively correlated with gestational age, but negatively correlated with ADHD (Figures 2F,G). Night waking was negatively correlated with age (Figure 2H). Parasomnia was positively correlated with ASD (Figure 2I). SDB was positively correlated with age and cognitive developmental delay (Figures 2J,K).

**Table 3:** Correlation between epidemiological and neurodevelopmental status and CSHQ.

	**Age**	**Gestational age 1^#^**	**Gestational age 2^#^**	**Gross motor**	**Fine motor**	**Speech**	**Cognition**	**Social emotion**	**ADHD**	**ASD**
Total sleep disturbance score	0.038	0.177^*^	0.282^**^	0.016	−0.055	−0.091	0.088	0.132	−0.085	0.161^*^
Bedtime resistance	−0.037	0.198^**^	0.257^*^	0.037	0.017	−0.117	0.067	0.140	−0.153	0.134
Sleep onset delay	0.057	0.111	−0.067	−0.093	−0.013	−0.157^*^	−0.094	−0.179^*^	0.118	−0.029
Sleep duration	0.089	0.079	−0.041	0.012	0.039	0.022	0.048	−0.002	−0.049	0.162
Sleep anxiety	−0.044	0.166^*^	0.151	0.115	−0.022	−0.104	−0.001	0.055	−0.183^*^	0.050
Night wakings	−0.193^**^	0.048	0.026	−0.012	−0.051	0.069	0.135	−0.030	−0.038	−0.027
Parasomnia	−0.038	0.057	0.075	−0.017	−0.077	0.021	0.128	0.128	−0.031	0.195^*^
Sleep disordered breathing	0.149^*^	0.060	0.193	0.002	−0.049	−0.041	0.175^*^	0.105	0.023	0.138
Daytime sleepiness	0.135	0.136	0.165	0.022	−0.076	−0.109	0.015	0.166	−0.081	0.083

All data are presented as correlation coefficients. ADHD, attention deficit-hyperactivity disorder; ASD, autism spectrum disorder; CSHQ, the Children's Sleep Habits Questionnaire. #Gestational age 1: All patients, divided into extremely preterm, very preterm, moderate preterm, late preterm, and full-term for correlation analysis. Gestational age 2: Only the preterm group, divided into extremely preterm, very preterm, moderate preterm, and late preterm.

** *p* < 0.01;

* *p* <0.05. Bold font indicates statistical significance.

Because previous studies showed that preterm birth is related to increased risks of sleep disordered breathing, and the risks increased with the decreasing gestational age (13, 14), we further selected the preterm group only for correlation analysis (Table 3, column Gestational age 2). Gestational age was still positively correlated with total sleep disturbance score and bedtime resistance. No significant correlation between gestational age and sleep disordered breathing was noted in our patients.

**Figure 3:**
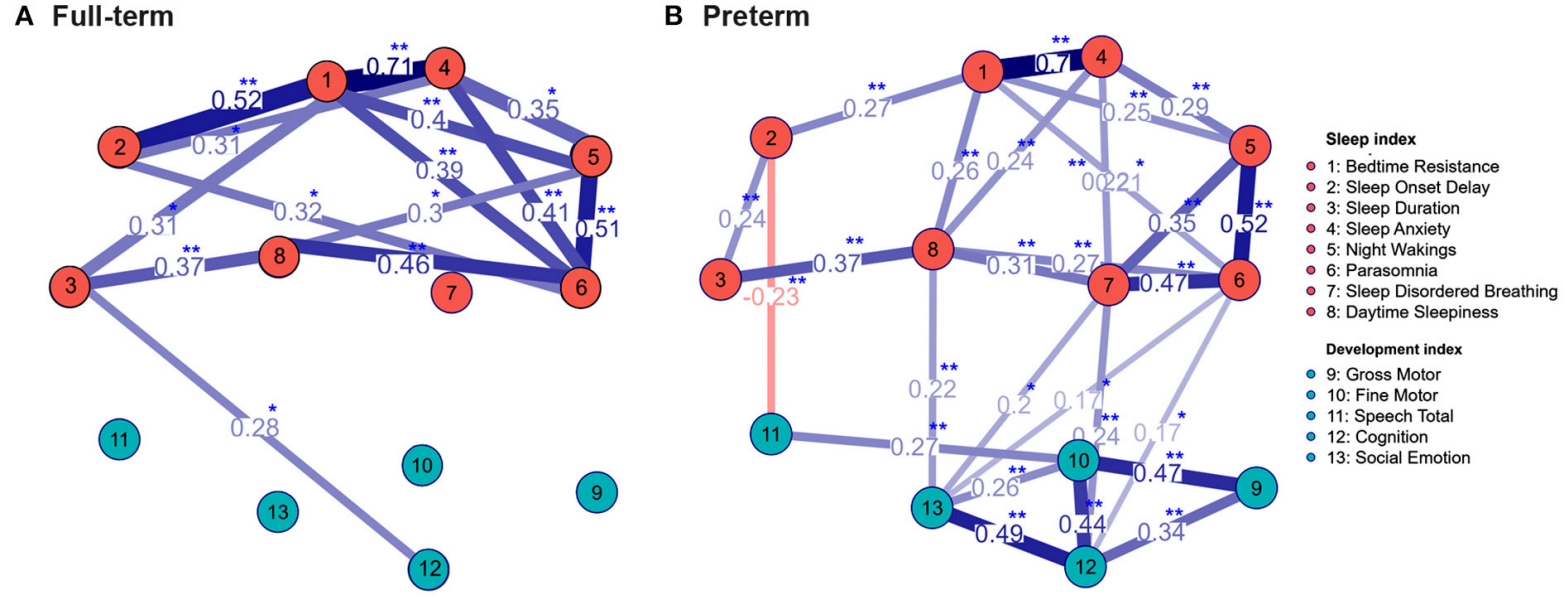
The community network structure of sleep problems and neurodevelopment status in the full-term **(A)** and preterm **(B)** group. The correlation coefficients between total 13 indexes with significant interactions were labeled. Significant interactions between sleep problems and neurodevelopmental status were mostly noted in the preterm group. ^*^p < 0.05; ^**^p < 0.01 using Spearman correlation analysis.

We further used the community network analysis to evaluate the interaction strength between sleep problems and developmental status in the full-term and preterm groups (Figure 3). Significant interactions between sleep problems and developmental status were noted in the preterm group, including SDB and cognitive developmental delay, SDB and socio-emotional developmental delay, parasomnia and cognitive developmental delay, parasomnia and socio-emotional developmental delay, and daytime sleepiness and socio-emotional developmental delay. In addition, a negative correlation between sleep onset delay and speech developmental delay was noted. In the full-term group, only one significant interaction between sleep duration and cognitive developmental delay was noted.

## Discussion

Our study showed that age, gestational age, and developmental status were correlated with sleep disturbances in preschool children at the Child Development Center.

There were no significant differences in development between the full-term and preterm groups in our study. Developmental delay rates were 73.4% in the full-term group and 77.6% in the preterm group. This seemed inconsistent with the common notion that preterm children are at a higher risk of developmental delay (8). One possible explanation is that most of the preterm children in our study were late preterm (70.4%), which is similar to a previous epidemiological preterm survey in the United States (15). Nevertheless, although often deemed as “near term,” late preterm children are still at an increased risk of developmental delay, including the domains of cognition, language, and motor development (16). Another possible explanation is that our participants were enrolled from the Child Development Center, to which only children, full-term or preterm, suspected to have developmental delay were referred. The prevalence of developmental disabilities in preschool children here in Northern Taiwan was 11.36% (17). The high prevalence (overall, 73.4%) of developmental delay in this study is similar to the prevalence (87.9%) in a UK study from a non-profit organization supporting families of children with neurodevelopmental condition (2). Similar to the above-mentioned population survey (17), speech developmental delay was the most common, followed by motor developmental delay.

Regarding sleep disturbance, the present study showed that full-term children had higher rates of sleep problems, sleep onset delay and bedtime resistance than preterm children. Durankus et al. showed that preterm preschool children had higher total sleep disturbance scores than full-term children (1). The subscale scores between the preterm and full-term groups were similar in their study. The developmental status, except for autism and ADHD, was not described in their study. A longitudinal study also showed no differences in sleeping behavior between full-term and preterm children at 20 and 56 months of age (18). Developmental status was not assessed in their studies. In a recent study in China, preterm birth was not associated with sleep problems in children aged 3–6 years (19). Our participants, both the full-term and preterm groups, had higher rates of sleep problems, defined by the cut-off point 41, than the general preschool children (90.4%/88.8% vs. 60.7%/70.8%) (1). These inconsistent findings imply that prematurity may not be the sole risk factor for sleep disturbances in children. Developmental delay may also aggravate sleep problem in preschool children.

Previous studies investigating sleep disturbances in preterm and full-term children did not subcategorize preterm groups based on gestational age. In this study, as gestational age increased, the total sleep disturbance score, bedtime resistance, and sleep anxiety increased in preschool children (Table 3; Figure 2). More bedtime resistance and sleep anxiety in full-term children than the in preterm children are consistent with a Norwegian cohort study (9). Preterm children have an earlier sleep phase than full-term children, and this advance sleep phase can persist into adolescence (20). Therefore, compared with full-term children of the same age, preterm children's parents are more likely to report less bedtime resistance and sleep anxiety in their children.

In our study, as ages increased, night wakings decreased and SDB increased. In addition to the decreased night wakings with age in children with typical development, night wakings decreased with age, which is consistent with a previous study on children with neurodevelopmental conditions (2). During a survey of SDB in preschool children in the United States, a similar age-dependent trend of prevalence of SDB was shown, although statistically insignificant (21). In fact, two peak periods of obstructive sleep apnea in children were noted: the first peak from 2 to 8 years, related to enlarged adenoid and/or tonsils, and the second during adolescence, related to weight gain (22).

In our study, children with speech and socio-emotional developmental delay had fewer problems with sleep onset delay. An association between sleep and speech development has been reported (23–26). Good sleep–wake consolidation in the first 2 years of life may foster language learning (23). Good sleep quality and napping may be critical for language acquisition and the generalization of word meanings (24). This beneficial effect of sleep on speech and language development may be associated with sustained attention (25). In addition, infants who developed a circadian rhythm by the third month of age exhibited better language development from 12 to 24 months of age than those who did not, indicating a cross-domain developmental cascade between sleep and language during the first 2 years of life (26). Our paradoxical finding showed that children with speech and socio-emotional developmental delay showed less delayed sleep onset than children with typical development, warranting further investigation, with an increased sample size, to confirm this finding.

In our study, children with intellectual disabilities were more likely to have SDB than children with typical development. Obstructive sleep apnea is prevalent in up to 95% of individuals with intellectual disabilities such as Down syndrome, Prader–Willi syndrome, Pierre–Robin sequence, and 22q11 deletion syndrome (27). During a survey of sleep problems in children with mild to profound intellectual disability in the Netherlands, the most common sleep problem was bedtime resistance (62.4%); SDB (mouth breathing when asleep, 43.8%; loud snoring, 22.4%) was also common (28). Children with severe sleep problems have more severe intellectual disabilities and more daytime behavioral problems, such as aggression, non-compliance and hyperactivity (28).

Patients with ADHD may have sleep problems including short sleep, delayed sleep onset, bedtime resistance, insomnia, fractured sleep, restless leg syndrome/periodic limb movement disorder, SDB, and daytime sleepiness (5). In addition to ADHD, comorbidities and medication may contribute to sleep problem. Thirty-three percent of patients with ADHD had an anxiety disorder, and 33% had dysthymic disorder or major depression (29). Different subtypes of ADHD and comorbidities present with different sleep problems (29). Interestingly, our study showed that children with ADHD had less sleep anxiety, as reported by their parents, which needs further investigation.

Common sleep problems in children with ASD include insomnia, characterized by difficulty falling asleep and/or maintaining sleep, reduced sleep duration, circadian sleep disturbance, and parasomnias (30). Sleep disturbances in children with ASD are significantly associated with irritability, inattention, and physical aggression (30). Both comorbid conditions (such as anxiety, fear, ADHD, sensory attributes, repetitive review of activities etc.) and biological reasons (abnormal metabolism of melatonin, dysregulation of circadian clock) predispose individuals to sleep disturbances (5). In our study, children with ASD had more sleep disturbances and parasomnias, which is consistent with previous studies.

In children with developmental delay, sleep disturbances are associated with multifactorial influences, including intrinsic pathophysiological factors, physical and psychiatric comorbidities, and pharmacological and parental influences (31). In our study, the interactions between the development status and sleep problems were significant mostly in the preterm group (Figure 3), suggesting other non-development factors affect the sleep problems in the full-term group more than the developmental status.

As mentioned earlier, the interaction between development and sleep is bidirectional. Assessing sleep problems during child development evaluation and integrating sleep therapy in early intervention programs can not only mitigate sleep problems but also maximize developmental potential and reduce stress within the family (32).

The limitations of our study include the following: (1) lack of objective sleep tools such as polysomnography and actigraphy; since the CSHQ has inadequate validity compared to polysomnography and actigraphy, using CSHQ as the sole screening tool for sleep problems in children should be done cautiously. In addition, a cut-off score of 41 for sleep problems may not be applicable to Chinese children probably because of genetic or sociocultural differences (33). Nevertheless, compared with a Chinese control group in a previous study (33), our participants still had significant higher total sleep disturbance scores, implying that children with developmental delay had more sleep problems than the control groups. In addition, the CSHQ is readily accessible in clinics and can still be used as the first screening tool for sleep problem in children (2). Only some of the children (66%), suspected to have psychiatric problem, underwent psychological evaluation. Since psychological evaluation takes much more time than other domains of developmental evaluation, we only referred children suspected of having psychological problems at the Child Developmental Clinic.

## Conclusion

Our results demonstrated an increased prevalence of sleep disturbances in children at the Child Development Center, and the sleep problems were associated with multiple factors, including age, gestational age, and developmental status. Neither prematurity nor developmental status is the sole risk factor for sleep problems in the preschool children with possible developmental delay. Evaluating sleep problems in preschool children should include both gestational age and developmental status. Therefore, during the evaluation of children with possible developmental delay, inquiring about their sleep quality and habits is strongly recommended. In addition, mitigating sleep problems in preschool children with developmental delay enhances the efficacy of early intervention programs.

## Data availability statement

The datasets presented in this article are not readily available because the data are not publicly available due to privacy or ethical restrictions. Requests to access the datasets should be directed to M-TY, mingtao.yang.tw@gmail.com.

## Ethics statement

The studies involving human participants were reviewed and approved by the Research Ethics Review Committee of the Far Eastern Memorial Hospital, New Taipei City, Taiwan (FEMH 110086-F). Written informed consent to participate in this study was provided by the participants' legal guardian/next of kin.

## Author contributions

C-MK and J-RL collected all samples and wrote the manuscript. S-HC performed statistical analysis and drew figures. J-SL reviewed the references. M-TY designed the study and revised the manuscript. All authors read and approved the final manuscript.

## Funding

This work was supported in part by the Far Eastern Memorial Hospital (FEMH-YZU-2021-001 and FEMH-2021-C-094).

## Conflict of interest

The authors declare that the research was conducted in the absence of any commercial or financial relationships that could be construed as a potential conflict of interest.

## Publisher's note

All claims expressed in this article are solely those of the authors and do not necessarily represent those of their affiliated organizations, or those of the publisher, the editors and the reviewers. Any product that may be evaluated in this article, or claim that may be made by its manufacturer, is not guaranteed or endorsed by the publisher.
